# Atypical Findings of Guillain-Barré Syndrome in Children

**Published:** 2012

**Authors:** Parvaneh KARIMZADEH, Mohammad Kazem BAKHSHANDEH BALI, Mohammad Mehdi NASEHI, Seyyed Mohaddeseh TAHERI OTAGHSARA, Mohammad Ghofrani

**Affiliations:** 1 Professor of Pediatric Neurology, Pediatric Research Center, Shahid Beheshti University of Medical Sciences (SBMU), Tehran, Iran; 2 Professor of Pediatric Neurology, Pediatric Neurology Department, Mofid Children Hospital, Faculty of Medicine, Shahid Beheshti University of Medical Sciences, Tehran, Iran; 3 Fellow of Pediatric Neurology, Pediatric Neurology Research Center, Shahid Beheshti University of Medical Sciences (SBMU), Tehran, Iran; 4 Associate Professor of Pediatrics, Department of Pediatrics, Mazandaran University of Medical Sciences, Sari, Iran; 5 General Physician, Tehran University of Medical Sciences, Tehran, Iran

**Keywords:** Guillain-Barre syndrome, Polyneuropathy, Cerebrospinal ﬂuid

## Abstract

**Objective:**

Guillain-Barre syndrome (GBS) is an immune-mediated polyneuropathy that occurs mostly after prior infection. The diagnosis of this syndrome is dependent heavily on the history and examination, although cerebrospinal ﬂuid analysis and electrodiagnostic testing usually confirm the diagnosis.

This is a retrospective study which was performed to investigate the atypical features of GBS.

**Materials & Methods:**

Thirty three patients (21/63.6% males and 12/36.4% females) with GBS were retrospectively studied and prospectively evaluated at the Child Neurology institute of Mofid Children Hospital of Shahid Beheshti University of Medical Sciences between May 2011 and September 2012.

**Results:**

The mean age was 5.4 years (range, 1.5-10.5).Twenty one patients (87.9%) had previous history of infections. Eight patients (24.2%) admitted with atypical symptoms like upper limb weakness (3%), ptosis (3%), neck stiffness (3%), inability to stand (proximal weakness) (9.1%), headache (3%) and dysphagia (3%).According to disease process, weakness was ascending in 26 (78.8%), descending in 5 (15.2%) and static in 2 (6.1%) patients. Cranial nerve involvement was found in 8(24.3%) children, most commonly as facial palsy in 3 (9.1%).

**Conclusion:**

In this study, 24.3% of our patients presented with atypical symptoms of GBS as upper limb weakness, ptosis, neck stiffness, inability to stand (proximal weakness), headache and dysphagia.

## Introduction

An acute inflammatory demyelinating polyneuropathy (AIDP) which has autoimmune basis affecting the peripheral nervous system is called Guillain –Barré syndrome (GBS). Nowadays Guillain–Barré syndrome is the most common cause of acute neuromuscular paralysis in the world. A French physician first described a variant of this syndrome in 1859, so it is sometimes called Landry’s paralysis. After the French physicians, this syndrome was detailed by Guillain, Barré and Strohl, in 1916. The disease has become well-known internationally under the name of Guillain Barré Syndrome (1,2).

The highest incidence of GBS is in two ends of the human life in early and late adulthood. The disease affects young adults greater than the elderly with a slightly higher male preponderance with a ratio of 1.25-1.5:1 (3). In Iran, the incidence of GBS has been reported between 1.5 and 3.4/100,000 versus 0.4 and 0.6/100,000 in western countries (4,5). 

The disease is assumed to be autoimmune and operated by a preceding infection, most of the time respiratory or gastrointestinal infections. Generally infections by microorganisms such as Campylobacter jejuni, CMV, Mycoplasma pneumonia, or inﬂuenza virus exist several weeks prior to approximately two thirds of GBS cases (6).

Epitopes on the surface of peripheral nerves (gangliosides, glycolipids) resemble some infectious agents’ surface epitopes so the immune system attacks peripheral nerves erroneously (6). GBS usually begins abruptly with distal, relatively symmetrical onset of paresthesias. Progressive limb weakness starts simultaneously or immediately after the sensory discomfort. Affected patients are able to remember the apparent onset of the disease symptoms. With a rapid progression, fifty percent of patients overtake clinical nadir by 2 weeks and more than 90% by 4 weeks (7). 

Miller Fisher syndrome, acute inflammatory demyelinating polyradiculoneuropathy (AIDP), acute motor axonal neuropathy (AMAN) and acute motor and sensory axonal neuropathy (AMSAN) are considered as four principal subtypes of this syndrome. Oculomotor dysfunction, ataxia and areflexia are triad symptoms of the Miller Fisher syndrome (8). Symptoms such as: progression of motor dificit, relative sensory symptoms or signs likes: elevated levels of protein in the cerebrospinal fluid and electrodiagnostic features such as nerve conduction slowing are criteria for GBS diagnosis (9). Temporal dispersion, significantly slow conduction velocities and prolonged distal and F-wave latencies are demyelinating findings of electrodiagnostic testing (EDX) of GBS. Electrodiagnostic testing results such as conduction block, temporal dispersion and nonuniform slowing of conduction velocities are useful in the diagnosis of the disease because they are specifications of immune-mediated demyelination (10).

Patients with the confirmed diagnosis of GBS will be treated with plasmapheresis or intravenous immunoglobulin (IVIG). Some patients should be under respiratory support when needed. Ventilator support is the requirement of approximately twenty five percent of GBS cases, particularly patients with more rapid motor weakness progression. The efficacy of plasmapheresis and IVIg in the treatment of GBS has been equal. Intravenous immunoglobulin (IVIG) is the treatment of choice of these patients because it is easier to use. Current recommendations corroborate the opinion that the steroid regimen has no more advantage when added to therapy, although this issue is controversial. Longitudinal gradual improvement will be the consequence of treatment in most patients in weeks to months. Affected patients with more aggressive onset tend to have more poorly recovery. Ten to twenty percent of these cases are complicated with a disabling motor deficit overall (11-13).

In this study, we evaluated patients with GBS series between 2011-2012, with the purpose of defining its atypical epidemiological, clinical, laboratorial findings and follow-up profile.

## Materials & Methods

This series of patients with Guillain-Barré syndrome were retrospectively studied and prospectively evaluated at the Child Neurology Institute of Mofid Children Hospital of Shahid Beheshti University of Medical Sciences between May 2011 and September 2012. The Child Neurology Institute of Mofid Children Hospital is a third level pediatric hospital that receives patients from throughout the country. The patients fulfilled the clinical criteria for Guillain-Barré syndrome (14). Based on the patient’s history, physical examination and paraclinic findings, the typical ascending form of Guillain-Barré syndrome was characterized by progressive, symmetric, ascending flaccid paresis with areflexia. The atypical presentation group of Guillain-Barré syndrome was characterized by localized or regional involvement of the motor and sensory axons of the peripheral nerves and the autonomic nervous system (15,16). Acute inflammatory demyelinating polyradiculoneuropathy (demyelination and axonal forms), acute motor-sensory axonal neuropathy and acute motor axonal neuropathy classified as the typical ascending GBS. Atypical presentation included prominent cranial nerve involvement, Miller Fisher syndrome, Bickerstaff brainstem encephalitis, pharyngo-cervical-brachial and polyneuritis cranialis and others, which included acute pandysautonomia and acute sensory neuropathy (17,18).

All patients were reviewed clinically by a child neurologist. The electrophysiology was performed at the neurophysiology section of Mofid hospital. Data was prospectively collected from all GBS cases and subsequently, cases were analyzed separately. The diagnosis was based on clinical features, lumbar puncture and electrophysiological findings as laid down in the well-established criteria. Onset of weakness, duration of weakness, associated or preceding events and progression of the disease were recorded. A detailed neurological examination was recorded in all patients. 

The nerve conduction velocity (NCV) studies were done within 24 hours of hospitalization. At least one motor and one sensory nerve were tested in upper and lower limbs. F-wave latencies were recorded if there was only mild slowing of nerve conduction velocity. 

Electromyography (EMG) was not performed in any patient. All confirmed GBS cases were followed up for 3 months after onset. 

## Results

During a period of 16 months, 33 children including 21 (63.6%) males and 12 (36.4%) females with the confirmed diagnosis of GBS were enrolled in this study. The mean age was 5.4 years (range, 1.5-10.5 years). Twenty one patients (87.9%) had a previous history of infection. The most common preceding events of the GBS occurred in winter and were respiratory infections (14 cases, 42.4%), followed by gastrointestinal infections (11 cases, 33.3%), dental infection (two cases, 6.1%), chicken pox (one case, 3%) and heart surgery (one case, 3%). In four other patients (12.1%) no triggering factor was found. The interval between these events and the onset of disabilities ranged from 3 to 15 days (mean, 9.5 days). The most common presenting symptoms were lameness that was seen in 10 (30.3%), gait imbalance in six (18.2%), lower limb weakness in five (15.2%), steppage gait in two (6.1%) and myalgia in two (6.1%) of the cases. 

Eight patients (24.2 %) were admitted with atypical symptoms like upper limb weakness (3%), ptosis (3%), neck stiffness (3%), inability to stand (proximal weakness) (9.1%), headache (3%) and dysphagia (3%). 

Lower limb deep tendon reflexes (DTR) were reduced or could not be detected in 27 (81.8%) where upper limb deep tendon reflexes (DTR) were reduced or could not be detected in 11 (33.3%) cases. According to the disease process, weakness was ascending in 26 (78.8%), descending in five (15.2%) and static in two (6.1%) patients. Cranial nerve involvement was found in eight (24.3%) children, most commonly as facial palsy in three (9.1%), followed by bulbar weakness (gag reflex abnormalities) in two (6.1%), abducence palsy in two (6.1%) and trigeminal palsy (ophthalmoplegia) in one (3%) patient. Five patients (15.2%) had sphincter dysfunction of which all were urinary incontinency. Nine patients (27.3%) complained of muscle pain and other sensory symptoms. A lumbar puncture was performed in all cases within two weeks of the onset of the illness. 

The CSF protein concentration was raised (>45 mg/dl) in 23 (69.6%) patients and the mean concentration was 59.8 mg/dl (range, 15-153 mg/dl). CSF pleocytosis was not found.

Nerve conduction velocity findings were as acute demyeliniting in 21 (63.6%), axonal in six (18.2 %) and absent F wave in six (18.2%) patients. Based on clinical and electrophysiological investigations, 29 (87.8%) of the cases were classified as AIDP, two (6.1%) of the cases were classified as CIDP and two (6.1%) as Miller Fisher syndrome (MFS). In three patients that were diagnosed as acute GBS, the cauda equine displayed hyperintense thickening in spinal MRI. Nine (27.2%) patients were admitted in PICU and ventilation support was needed for four (12.2%) of them. 

Twenty six patients received IVIG and four of them received intravenous methylprednisolone too. Four of the patients were treated with plasmapheresis after IVIG - corticosteroid failure and three cases were cured without any medication. The length of hospital stay ranged from 5 to 36 days (mean 7.5 days).

After treatment, 19 patients (57.6%) developed significant improvement of functional disability one month later, five of them (15.1%) ameliorated after 3 months and nine (27.3%) patients have not reached complete recovery till now. Among untreated children, two have waddling gait, five have inability to stand, one has steppage gait and two have limb lameness.

## Discussion

We have reported eight children who include 24.3% of the total patients who have presented with atypical symptoms of GBS as upper limb weakness (3%), ptosis (3%), neck stiffness (3%), inability to stand (proximal weakness) (9.1%), headache (3%) and dysphagia (3%). Jin Park et al. reported an 11-year-old girl with an atypical GBS presentation of generalized weakness, internal ophthalmoplegia (visual dimness, anisocoric pupils) and postural dizziness; The current study is consistent with the symptoms of a 10-year-old girl who was admitted with ptosis and weakness in our study (19).

A 1.5-year-old boy was referred to our hospital with neck stiffness and myalgia. Central nervous system infections were ruled out by CSF analysis and electrophysiologic investigation found acute demyelinating neuropathy as GBS. This is similar to the report of atypical GBS by Etem Pişkin et al. who diagnosed a 3-year-old girl who suffered from prominent neck stiffness and positive Kernig’s and Brudzinski’s signs. Muscle weakness increased significantly on the second day of hospitalization and the patient lost her ability to walk by the third day (20).

In our series of 33 children with the diagnosis of GBS, 87.8% had clinical and electrophysiological findings suggesting AIDP; 6.1% of the cases were classified as CIDP and 6.1% as Miller Fisher syndrome which is concordant with previous studies. 

In North America and Europe, Miller-Fisher syndrome has been found to account for approximately 5% of the GBS cases (8).

The male to female ratio was 1.75:1, while previous reports presented a ratio of 1.25-1.5:1 (21). Most authors described a discrete prevalence of GBS in males. On the other hand, van der Linden et al. reported no significant discrepancy between the two genders regarding the incidence of GBS (22). 

Twenty one patients (87.9%) had previous history of infections with a mean interval between these events and disability onset of 9.5 days. Two most common preceding events of the GBS were respiratory (42.4%) and gastrointestinal infections (33.3%). Generally, more than two-thirds of GBS cases have a past medical history of acute illness, most commonly infection of the respiratory tract or gastrointestinal system which have been eliminated since the neuropathic symptoms initiated. In several large studies, the time duration between the precedent infection and the symptoms of GBS differs from 1 to 3 weeks which is sometimes longer. In many large studies, the average of 11 days has been reported. Precedent respiratory and gastrointestinal infectious symptoms encompassed 17% to 38% of the patients with GBS in a study conducted by Winer et al. (23). 

This study showed that the highest proportion of the syndrome was observed in the winter; however. The syndrome assumed to be sporadic with no significant disparity between seasons or months. This finding is in agreement with a study performed by Barzegar et al. which showed a relationship between the high frequency of upper respiratory tract infections within the cold season and the high incidence of GBS syndrome in winter (24). A study from China demonstrated that campylobacter jejuni infections in summer might create epidemics of this syndrome. The seasonal incidence of 40% has been reported for GBS in cold seasons with the maximum peak in February in a study carried out in Saudi Arabia (24,25). The weakness was ascending in 78.8% of the patients; however, in 15.2% the initial symptoms involved the upper limbs. The involvement of cranial nerves occurred in 24.3% of the patients, VII nerve being the most attacked, usually unilateral; which was different to that found in the literature (26). 

The pattern of cranial nerve involvement in a study conducted by van der Linden et al. on the clinical and epidemiological aspects of Guillain-Barré syndrome in children was bilateral facial nerve paralysis in 55.7% (22).

The mean recovery period in children reported in the literature was about 50 days, which is rather a short time. Total time for recovery of GBS is shorter in younger patients compared with adults. Nine patients (27.3%) persisted with sequels after a period of 16 months follow-up, which is in agreement with that

found in the literature (27,28). However, the sequel rate reported here is higher than some studies, because Mofid hospital is a third level pediatric hospital that receives patients from throughout the country including those without complete recovery.
